# The Appropriateness of Language Found in Research Consent Form Templates: A Computational Linguistic Analysis

**DOI:** 10.1371/journal.pone.0169143

**Published:** 2017-02-01

**Authors:** Alexander Villafranca, Stephanie Kereliuk, Colin Hamlin, Andrea Johnson, Eric Jacobsohn

**Affiliations:** University of Manitoba, Department of Anesthesiology and Perioperative Medicine, CR31-42 369 Tache Ave, Winnipeg, MB, Canada; University of Liverpool, UNITED KINGDOM

## Abstract

**Background:**

To facilitate informed consent, consent forms should use language below the grade eight level. Research Ethics Boards (REBs) provide consent form templates to facilitate this goal. Templates with inappropriate language could promote consent forms that participants find difficult to understand. However, a linguistic analysis of templates is lacking.

**Methods:**

We reviewed the websites of 124 REBs for their templates. These included English language medical school REBs in Australia/New Zealand (n = 23), Canada (n = 14), South Africa (n = 8), the United Kingdom (n = 34), and a geographically-stratified sample from the United States (n = 45). Template language was analyzed using Coh-Metrix linguistic software (v.3.0, Memphis, USA). We evaluated the proportion of REBs with five key linguistic outcomes at or below grade eight. Additionally, we compared quantitative readability to the REBs’ own readability standards. To determine if the template’s country of origin or the presence of a local REB readability standard influenced the linguistic variables, we used a MANOVA model.

**Results:**

Of the REBs who provided templates, 0/94 (0%, 95% CI = 0–3.9%) provided templates with all linguistic variables at or below the grade eight level. Relaxing the standard to a grade 12 level did not increase this proportion. Further, only 2/22 (9.1%, 95% CI = 2.5–27.8) REBs met their own readability standard. The country of origin (DF = 20, 177.5, F = 1.97, p = 0.01), but not the presence of an REB-specific standard (DF = 5, 84, F = 0.73, p = 0.60), influenced the linguistic variables.

**Conclusions:**

Inappropriate language in templates is an international problem. Templates use words that are long, abstract, and unfamiliar. This could undermine the validity of participant informed consent. REBs should set a policy of screening templates with linguistic software.

## Introduction

In order for research participants to provide valid consent they must be able to understand study-related information [1]. Research consent forms are a primary means of providing this information and acquiring informed consent. In English-speaking countries (Australia, Canada, New Zealand, South Africa, the United Kingdom, and the United States of America), 70–90% of adults have completed upper secondary education [2]. However, national literacy assessments demonstrate that 42–53% of adults in these same countries have deficient literacy skills [3,4], estimated to correspond with a grade eight reading level or lower [5]. In response, health organizations including the World Health Organization [6] and the American Medical Association [7] recommend that documents written for patients should use language not exceeding a grade six to eight level. However, studies find that the majority of clinical research consent forms are written at higher grade levels [8–13].

REBs endeavor to promote appropriately written consent forms by providing researchers with consent form templates. These templates include a description of the information that should be disclosed in a research consent form, and provide recommended language to use in disseminating specific information. This language is important because it normally involves information that is either germane or difficult to understand. Researchers are also likely to use this language verbatim on consent forms.

Two investigations demonstrated that recommended language in templates from the USA have poor readability [14, 15]. Before we can properly recommend a solution, important limitations of these studies must be addressed. First, these studies only examined quantitative readability, a metric based on word and sentence length. If we assess recommended language solely by quantitative readability, we gain an incomplete picture of its appropriateness. Researchers should analyze recommended language using other linguistic variables such as word familiarity and imagability, which provide different information and are important predictors of the understanding of a text [16]. Second, the two studies also calculated quantitative readability using Microsoft Word. This software has been shown to overestimate quantitative readability and to have serious inconsistencies [17]. Third, these studies also did not identify if the poor quantitative readability was due to long words, or sentences, or both [16], and therefore could provide little guidance on how templates could be improved. Finally, this previous research only examined templates from the USA. Further research is therefore necessary to improve the measurement of the recommended language in templates and to extend the generalizability of the results. This study measured five key linguistic variables of templates from six English-speaking countries, using academically developed, peer-reviewed linguistic software [18].

## Methods

### Design

We conducted a computational linguistics analysis of REB templates that were publicly available online. Local REB approval was not required as no human or animal research subjects were involved, and texts examined were in the public domain.

### Data sources and extraction

The selection criteria for templates were that they must be documents supplied by REBs to researchers; outline required disclosure elements and provide recommended wording in the form of example sentences or sentence fragments exceeding three words; pertain to clinical research; be intended for research on competent adults; and not be specialized documents intended only for a certain type of research (e.g. genetic studies, blood banks studies). Many REBs provide multiple reference documents, but we reviewed only the most general documents. These documents apply to the largest number of studies, and researchers are therefore most likely to access these documents for guidance.

The population of medical schools in Australia, New Zealand, Canada, South Africa, the United Kingdom, and the United States was identified using the websites of national accreditation bodies (Table 1). From this, we excluded any medical schools that taught exclusively in another language. Because of the much larger number of medical schools in the Unites States, we included a geographically stratified subsample in our study. All included medical schools are listed in S1 Table. A total of 124 REB websites were reviewed for templates.

**Table 1:** Inclusion of medical schools

Country	Total accredited medical schools	Source/Accrediting body	Schools teaching in non- English languages	Schools reviewed for REB templates
**Australia**	19	Australian Medical Council	0	21
**Canada**	17	Committee on Accreditation of Canadian Medical Schools (The Association of Faculties of Medicine of Canada)	3	14
**New Zealand**	4	Medical Council of New Zealand	0	4
**South Africa**	8	Health Professions Council of South Africa	0	8
**United Kingdom**	34	General Medical Council, Medical Schools Council, UK	0	34
**United states**	141	Liaison Committee on medical Education supported by the Association of American Medical Colleges and the American Medical Association)	0	45

We selected the sub-sample of schools from the USA by dividing the country into the nine census sub-regions (i.e. “divisions”) recognized by the US census bureau [19], and then randomly selecting five medical schools from each region. For REBs that provided publicly accessible templates, we extracted the recommended language and prepared the text for computational linguistic analysis using a standardized data reduction protocol.

### Data analysis

A quantitative readability score was measured using the accepted Flesch-Kincaid quantitative readability grade level calculation, which is based on average word and sentence length [18]. Additionally, the individual components of quantitative readability (word and sentence length), and two other linguistic variables (word familiarity and imagability) were individually assessed. Word familiarity pertains to how familiar the involved words would be to a lay population [18], and average word imagability to how easy it would be for a lay population to visualize the involved words [18]. These linguistic measures were calculated using Coh-Metrix (v.3.0, University of Memphis, USA), an academically developed and peer-reviewed computational linguistics software package [18]. Statistical analysis was done using SAS (v.9.2, SAS Institute, Carey, USA). Coh-Metrix provides linguistic norms for different types of text, including science writing [18]. These norms are further divided into different grade level groupings from kindergarten to grade twelve. The norms for each grade level grouping were derived from 300 excerpts of science texts written for this grade level grouping [18]. We used these science text norms to develop regression equations allowing us to convert the raw scores of the language measures into their grade level equivalents (Table 2). We did this to increase the interpretability of the results, and to allow us to compare template language measures to specific grade level standards. Grades were allowed to range from zero to positive infinity.

**Table 2:** Regression equations to calculate language variable grade levels.

*Language variable (original units)*	*Grade level equivalent*	*R-squared*
***Sentence length (x = words/sentence)***	*y = 1·1675x - 8·4861*	0*·*98
***Word length (x = syllables/word)***	*y = 28·644x - 34·572*	0*·*99
***Word familiarity (x = arbitrary units*, *0–700)***	*y = -0·5718x + 333·94*	0*·*98
***Word imagability (x = arbitrary units*, *0–700)***	*y = -0·4147x + 184·77*	0*·*81

For the statistical analysis, the discrepancies between the observed template grade levels and a grade eight standard were calculated. This standard was selected as it is recommended by several influential health organizations such as the World Health organization, the American Medical Association, and the National Health and Medical Research Council [6, 7, 20]. It is also the standard most commonly recommended by REBs in the United States [14]. Furthermore, some health literacy experts have asserted that the grade eight reading level demarcates the boundary between low literacy and literacy [5, 21]. The main outcome was the proportion of REBs providing templates with all language outcomes equal or less than a grade eight standard. However, we conducted a sensitivity analysis to ensure that this proportion was similar with a less defensible but more liberal grade level (grade twelve).

Additionally, we used an intercept only MANOVA model to determine if the templates on average had linguistic variables deviating significantly from a grade eight level. This model tests whether the discrepancies from grade eight observed for each of the variables are significantly different from zero. If the REB provided their own recommended readability grade level either in their template or on their website, the REB was considered to have a local readability standard. To determine if the template’s country of origin or the presence of a local REB readability standard influenced the discrepancy magnitude, we used a second MANOVA model that included both predictors. For this comparison, we grouped templates from New Zealand and Australia together due to the small number of medical schools in the former. Pair-wise contrasts were used for this model, and a false discovery rate [22] of 5% was set to control for family-wise error. Where possible, we compared template quantitative readability to the magnitude of the local readability standard using a two-tailed, paired t-test. Confidence limits for all proportions were calculated using the exact binomial method (Clopper-Pearson).

## Results

**Fig 1:**
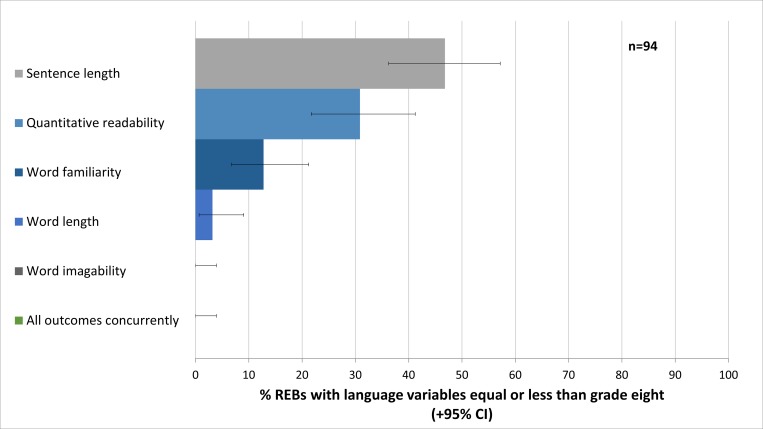
The proportion of research ethics boards providing language variables equal or less than the grade eight standard.

Of 124 REBs websites accessed, 94 (75.8%, 95% CI = 67.30–83) had a publicly available template. Fig 1 shows the proportion of REBs providing templates with linguistic variables equal or less than the grade eight level. Of note, none of the 94 REBs with publicly available templates had all measures equal or less than the grade eight standard (0%, 95% CI = 0–3.9%). This proportion remained zero even if the language standard was relaxed to the grade twelve level.

**Fig 2:**
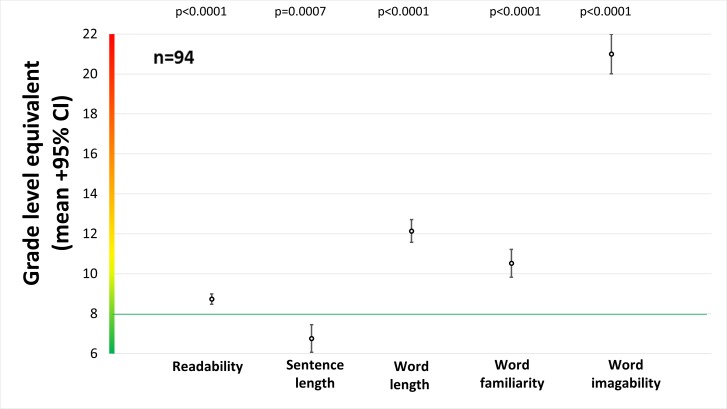
Average grade level equivalents for all language variables.

In Fig 2, we compare the grade level of template recommended language to a grade eight standard. All linguistic variables deviated significantly from this standard, with word length, familiarity, and imagability all higher than grade eight, while sentence length was lower than grade eight.

**Fig 3:**
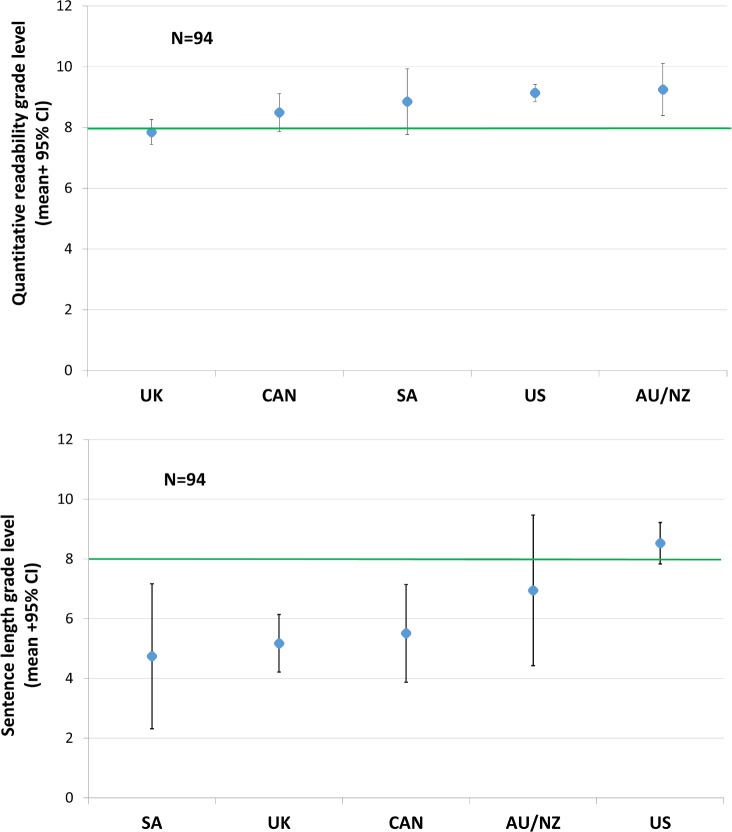
Grade levels for quantitative readability and sentence length, stratified by country of origin.

The country of origin (DF = 20, 177.5, F = 1.97, p = 0.01), but not the presence of an REB-specific standard (DF = 5, 84, F = 0.73, p = 0.60), influenced the discrepancies between template linguistic variables and a grade eight standard. However, post-hoc testing with multiple correction adjustment showed that only the discrepancies for quantitative readability (DF = 4, F = 4.32, p = 0.003) and sentence length were affected by country of origin (DF = 4, F = 3.70, p = 0.01). The templates from the UK had significantly better quantitative readability than those from Australia/New Zealand (DF = 1, F = 13.72, p = 0.0004) and the USA (DF = 1, F = 16.42, p<0.0001, Fig 3), while the templates from the US had significantly worse sentence length than those from the UK (DF = 1, F = 15.83, p<0.0001, Fig 3).

Twenty-two REBs provided local quantitative readability standards (median = 8, range = 6–8). Of these, only two (9.1%, 95% CI = 2.5–27.8) met their own standard. These local standards were exceeded by an average of 1.33 grade levels (p<0.0001).

## Discussion

This study shows that not one of the 94 templates analyzed contained recommended language at or below the recommended grade level. In fact, none of the templates even had language at or below a less acceptable grade 12 level. REBs from all six countries examined were equal in their poor performance, making inappropriate language in templates an international problem. Unexpectedly, the presence of an endorsed local quantitative readability standard did not improve any of the linguistic variables. As a result, most of the REBs that had set a local readability standard failed to meet their own standard.

These results are concerning. While we believe that these templates could be a powerful tool for knowledge dissemination, the REBs’ recommendation of complex language places them at risk of serious criticism. Researchers who use the recommended language in templates verbatim will create consent forms with key sentences that involve inappropriately complex language. Further, researchers who use the sophistication of recommended language as a guide are likely to create new sentences that contain inappropriately complex language. Research demonstrates that some participants have a level of understanding about the purposes and risks of the trial that compromises their consent [23–25]. Patients themselves sometimes feel that the study consent form has not given them a satisfactory level of understanding [26, 27]. The promotion of consent forms with inappropriately complex language through the use of poorly designed templates may contribute to these problems.

A lack of understanding is especially concerning for research participants with low literacy. These individuals may be at a greater risk of harm since their reduced understanding may result in lower compliance with study protocols. This is supported by research demonstrating that individuals with low literacy have poorer compliance with medical recommendations [28], are up to three times more likely to experience poor health outcomes [29], including risk of hospitalization, medication mismanagement, and death [29–32] than individuals who have high literacy. A lack of compliance with study protocols would also have implications beyond individual participants. It could affect the fidelity of any study interventions and thereby undermining the external validity of the study findings.

This study reveals that previous investigations [14, 15] have overestimated the appropriateness of recommended language in templates by not considering metrics beyond quantitative readability. While readability was less than one grade level above the grade eight standard, the word length, familiarity and imagability exceeded the standard by four, two, and thirteen grade levels, respectively. This analysis also reveals that templates are inappropriate in ways beyond the quantitative readability metric, as they used words that are lengthy, abstract, and unfamiliar.

This study can provide further guidance to REBs in improving their templates. Recommended language in templates generally involved sentences of acceptable length. This is likely attributable to the REBs frequent use of headers and bullet points, and the fact that we were often analyzing sentence fragments. Although reducing sentence length would improve quantitative readability, it would fail to address the main issue with templates. This study shows that the poor quantitative readability of recommended language in templates is due to word choice. The proper solution is to modify the vocabulary used, making it more familiar, short, and easy to visualize. An example of overly complex text that was present in one of the examined templates is shown in S2 Table, alongside a modification that greatly improves its linguistics.

Yet simply improving templates alone may not eliminate consent forms with poor language. Several other investigators have shown that clinical research consent forms used in REB approved studies are often poorly worded [9–13, 33]. This shows that some REBs are also not appropriately vetting the language used in the research consent forms that they review.

To improve the language in both templates and research consent forms, we propose that REBs routinely screen these document types using linguistic software. This process would be simple, inexpensive and could mitigate the problems identified in this study. An analogous process is currently see in the routine screening of submitted manuscripts for plagiarism [34].

Several limitations of this study merit discussion. We only evaluated the main template from each REB. Many REBs provide multiple reference documents, including required elements for consent forms, assent form templates, and templates for both generic and special circumstances. Due to software limitations, we were also unable to include three French language medical schools from Canada. The quality of templates from REBs with either password-protected websites or invalid web links (n = 14) could not be determined. Thus, the generalizability of our findings to these schools and to other countries is unknown. We also did not include linguistic variables that examine other important features of text, such as flow, content overlap, or sequencing of information within a document [18]. However, researchers use those variables to quantify complete texts. Recommended language in templates involves a series of unconnected sentences, and thus evaluating template text on the basis those variables would unfairly disadvantage the materials. The ease with which patients can read consent forms is also affected by the legibility (i.e. visual clarity) of the text, which was beyond the scope of this study. Factors influencing legibility include font typeface, size, color, contrast, text spacing, and text blocking [35]. Therefore, while improving the template wording may be necessary to improve patient understanding, this action alone may not be sufficient.

This is the most widespread study of templates to date. It uses an assessment of template recommended language that is both more comprehensive and more rigorous than assessments in previous investigations. It also provides guidance on how REBs should modify this template language to be more appropriate for patients.

In conclusion, inappropriate recommended language in templates is a ubiquitous international problem. Previous studies [8–15] together with our current findings provide strong evidence that some REBs are inadvertently promoting consent forms written at a level that many research participants find difficult to understand. To improve consent form language REBs should set a policy of screening their templates with linguistic software. Ensuring that templates contain appropriate recommended language would likely improve consent forms that are based on these templates. REBs should also consider screening the consent forms that they review, or insisting that researchers submit proof that the consent forms were screened prior to their submission. This would better accommodate individuals with poor health literacy by increasing the likelihood that they provide valid informed consent and by better safeguarding their welfare.

## Supporting Information

S1 Table List of all medical school websites that were screened for consent form templates. (DOCX)

S2 Table Examples of overly complex text and how it could be modified to improve its linguistic variables. (DOCX)
