# Supplementary material for: The Appropriateness of Language Found in Research Consent Form Templates: A Computational Linguistic Analysis
Source: PLoS One. 2017 Feb 1;12(2):e0169143. doi: 10.1371/journal.pone.0169143 (PMC5287453; doi:10.1371/journal.pone.0169143)
Supplement: S1 Table — (DOCX) [file pone.0169143.s001.docx]

| **Country** | **Universities included** |
| --- | --- |
| Australia | 1. Bond University 2. Flinders University 3. Deakin University 4. Griffith University 5. James Cook University 6. Monash University 7. Australian National University 8. University of Adelaide 9. University of Melbourne 10. University of New South Wales 11. University of Newcastle 12. University of New England 13. University of Notre Dame Australia, Sydney & Fremantle 14. University of Queensland 15. University of Sydney 16. University of Western Australia 17. University of Tasmania 18. University of Western Sydney 19. University of Wollongong |
| Canada | 1. Dalhousie University 2. McGill University 3. McMaster University 4. Memorial University of Newfoundland 5. Northern Ontario School of Medicine 6. Queen's University 7. University of Alberta 8. University of British Columbia 9. University of Calgary 10. University of Manitoba 11. University of Ottawa 12. University of Saskatchewan 13. University of Toronto 14. Western University |
| New Zealand | 1. Auckland University Faculty of Medical Health & Sciences 2. University of Otago, Wellington 3. University of Otago, Christchurch 4. Otago School of Medical Sciences |
| South Africa | 1. Stellenbosch University, Faculty of Medicine and Health Sciences 2. Universiteit van Pretoria, Faculteit Geneeskunde 3. University of Cape Town, Faculty of Medicine 4. University of KwaZulu-Natal, Nelson R. Mandela School of Medicine 5. University of Limpopo, School of Medicine (MEDUNSA-campus) 6. University of the Free State, Faculty of Health Sciences 7. University of the Witwatersrand, Faculty of Health Sciences 8. University of Transkei, Faculty of Medicine and Health Sciences (Walter Sisulu University) |
| United Kingdom | 1. Aberdeen (University of), School of Medicine 2. Barts and The London School of Medicine and Dentistry, Queen Mary, University of London 3. Birmingham (University of), School of Medicine 4. Brighton and Sussex Medical School (BSMS) 5. Bristol (University of), Faculty of Medicine 6. Cambridge (University of), School of Clinical Medicine 7. Cardiff University, School of Medicine 8. Dundee (University of), Faculty of Medicine, Dentistry and Nursing 9. Edinburgh (The University of), College of Medicine and Veterinary Medicine 10. Exeter (University of), Medical School 11. Glasgow (University of), College of Medical, Veterinary and Life Sciences 12. Hull York Medical School 13. Imperial College School of Medicine, London 14. Keele University, School of Medicine 15. King's College London School of Medicine (at Guy's, King's College and St Thomas' Hospital) 16. Lancaster University, Faculty of Health & Medicine 17. Leeds (University of), School of Medicine 18. Leicester (University of), Leicester Medical School 19. Liverpool (University of), Faculty of Health and Life Sciences 20. London School of Hygiene and Tropical Medicine (Postgraduate Medical School) 21. Manchester (University of), Faculty of Medical and Human Sciences 22. Newcastle University Medical School 23. Norwich Medical School, University of East Anglia 24. Nottingham (The University of), Faculty of Medicine and Health Sciences 25. Oxford (University of), Medical Sciences Division 26. Plymouth University Peninsula Schools of Medicine and Dentistry 27. Queen's University Belfast, Faculty of Medicine and Health Sciences 28. Sheffield (The University of), School of Medicine 29. Southampton (University of), School of Medicine 30. St Andrews (University of), Faculty of Medical Sciences 31. St George's, University of London 32. Swansea University, School of Medicine 33. University College London, University College Medical School 34. Warwick (The University of), Warwick Medical School |
| United States of America | New England   1. Boston University School of Medicine 2. The Warren Alpert Medical School of Brown University 3. Frank H. Netter MD School of Medicine at Quinnipiac University 4. Harvard Medical School 5. University of Connecticut, School of Medicine   West North Central   1. Washington University in St. Louis, School of Medicine 2. Saint Louis University, School of Medicine 3. University of Iowa Roy J. and Lucille A. Carver College of Medicine 4. University of North Dakota School of Medicine and Health Sciences 5. Creighton University, School of Medicine   South Atlantic   1. University of South Carolina, School of Medicine 2. Morehouse School of Medicine 3. Eastern Virginia Medical School (EVMS) 4. University of North Carolina School of Medicine 5. Medical University of South Carolina College of Medicine   East South Central   1. University of Alabama, School of Medicine 2. Vanderbilt University School of Medicine 3. University of Kentucky, College of Medicine 4. University of Louisville, School of Medicine 5. University of Tennessee Health Science Center College of Medicine   West South Central   1. University of Arkansas for Medical Sciences College of Medicine (UAMS) 2. University of Texas Medical School 3. Baylor College of Medicine 4. Texas Tech University Health Sciences Center, School of Medicine 5. Texas A&M Health Science Center College of Medicine   Middle Atlantic   1. Ponce School of Medicine and Health Sciences 2. Rutgers, Robert Wood Johnson Medical School 3. San Juan Bautista, School of Medicine 4. New York Medical College 5. Cooper Medical School of Rowan University   East North Central   1. University of Cincinnati College of Medicine 2. Wayne State University, School of Medicine 3. Rush Medical College of Rush University Medical Center 4. Stritch School of Medicine Loyola University Chicago 5. Central Michigan University, College of Medicine   Mountain   1. University of Colorado, School of Medicine 2. University of Utah School of Medicine 3. University of Nevada, School of Medicine 4. University of Arizona, College of Medicine 5. University of New Mexico, School of Medicine   Pacific   1. Keck School of Medicine of the University of Southern California 2. Loma Linda University, School of Medicine 3. John A. Burns School of Medicine, University of Hawaii at Manoa 4. Stanford University, School of Medicine 5. University of Washington School of Medicine |
