# Supplementary material for: The Appropriateness of Language Found in Research Consent Form Templates: A Computational Linguistic Analysis
Source: PLoS One. 2017 Feb 1;12(2):e0169143. doi: 10.1371/journal.pone.0169143 (PMC5287453; doi:10.1371/journal.pone.0169143)
Supplement: S2 Table — (DOCX) [file pone.0169143.s002.docx]

| **Original Text** | **Possible revisions** | **Linguistics** | | |
| --- | --- | --- | --- | --- |
|  |  | **Measure** | **Original (grade level)** | **Revision (grade level)** |
| “I understand that all identifiable (attributable) information that I provide is treated as strictly confidential and will not be released by the investigator in any form that may identify me.” | I know that the researchers are storing information about me on paper and on a computer. If someone read these files, they might figure out who I am. However, the study staff will keep these files private. Paper files will be locked in a cabinet. Computer files will be protected with a password. The researchers will write a paper and give talks about the study. When they do, they will not mention my name or other things that would identify me. | Sentence length | 26.5 | 5.0 |
|  |  | Word length | 20.7 | 6.1 |
|  |  | Word familiarity | 13.4 | 7.3 |
|  |  | Word imagability | 36.6 | 8.6 |
|  |  | Quantitative readability | 18.9 | 6.2 |
|  |  | Average | 23.22 | 6.8 |
